# Non-invasive fractional flow reserve estimation in coronary arteries using angiographic images

**DOI:** 10.1038/s41598-024-65626-9

**Published:** 2024-07-08

**Authors:** Hadis Edrisnia, Mohammad Hossein Sarkhosh, Bahram Mohebbi, Seyed Ehsan Parhizgar, Mona Alimohammadi

**Affiliations:** 1 Department of Mechanical Engineering, K. N. Toosi University of Technology, Tehran, Iran; 2 Department of Mechanical Engineering, Sharif University of Technology, Tehran, Iran; 3 Rajaie Cardiovascular, Medical and Research Center, Iran University of Medical Sciences, Tehran, Iran

**Keywords:** Haemodynamics, Left anterior descending artery (LAD), Computational fluid dynamics (CFD), Virtual Fractional Flow Reserve (vFFR), Wall shear stress (WSS), Atherosclerosis, Cardiovascular diseases, Vascular diseases

## Abstract

Coronary artery disease is the leading global cause of mortality and Fractional Flow Reserve (FFR) is widely regarded as the gold standard for assessing coronary artery stenosis severity. However, due to the limitations of invasive FFR measurements, there is a pressing need for a highly accurate virtual FFR calculation framework. Additionally, it’s essential to consider local haemodynamic factors such as time-averaged wall shear stress (TAWSS), which play a critical role in advancement of atherosclerosis. This study introduces an innovative FFR computation method that involves creating five patient-specific geometries from two-dimensional coronary angiography images and conducting numerical simulations using computational fluid dynamics with a three-element Windkessel model boundary condition at the outlet to predict haemodynamic distribution. Furthermore, four distinct boundary condition methodologies are applied to each geometry for comprehensive analysis. Several haemodynamic features, including velocity, pressure, TAWSS, and oscillatory shear index are investigated and compared for each case. Results show that models with average boundary conditions can predict FFR values accurately and observed errors between invasive FFR and virtual FFR are found to be less than 5%.

## Introduction

Coronary artery disease (CAD) is the leading global cause of mortality, comprising around 30% of total deaths^1^. Coronary atherosclerosis stands out as a prevalent factor in the development of cardiovascular disease^2^. The accumulation of atherosclerotic plaque within the coronary arteries has the potential to obstruct blood flow, resulting in an inadequate blood supply to the tissues and organs downstream. This process significantly contributes to a range of cardiovascular issues, including the onset of conditions like coronary heart disease and myocardial infarction^3–5^. Invasive Fractional Flow Reserve (FFR) is a way of indicating the maximum blood flow beyond a stenosis section of an artery compared to the pressure in the aorta during a state of increased blood flow^6^. In addition, invasive FFR demands the insertion of a pressure wire to pass through the stenosis segment, to measure the pressure at the far end of the narrowed region usually 2–3 cm away^6,7^. Handling the pressure wire during this procedure carries inherent risks^7^, given the potential for harm to the vessel walls or activation of the plaque within the artery. Furthermore, the process involves the injection of adenosine or ATP to induce hyperemia within the patient’s distal microvascular system and this step can pose a potential hazard, particularly for patients who exhibit intolerance to adenosine^8–10^. Another drawback is that FFR cannot recognize the early vulnerable plaques^11^. Coronary computed tomography angiography (CTA) is a standard procedure for patients suspected of having CAD. Nevertheless, there have been reports indicating that more than 50% of cases where severe stenosis was detected through CTA were later found to be overestimated^12,13^. This leads to a substantial number of unnecessary referrals for invasive angiography. The application of CFD- simulations for numerical haemodynamic analysis is widely acknowledged as an established methodology for exploring the haemodynamic mechanisms of cardiovascular diseases and their future outcomes^5,14^. This is primarily due to its capacity to visually represent haemodynamic distribution within coronary stenosis in three dimensions^15–17^. Merging CTA and FFR achieves good accuracy for CFD simulations, but prolonged hours are necessary for geometry reconstruction and patient-specific simulation becomes vital. Hence, the combination of image segmentation algorithms and CFD has spurred rapid advancements in patient-specific modeling capabilities^18^. CFD has the potential to utilize parameters such as local pressure gradient, wall shear stress, flow velocity, and vorticity alterations downstream of arterial plaque in addition to disease future prediction and evolution^19^. An increasing body of evidence suggests that both in idealized scenarios and patient-specific models, the regional Wall Shear Stress (WSS) and WSS-based indices have a crucial and foundational impact on the first stages and advancement of atherosclerosis, with low and oscillatory WSS being correlated with the site of atherosclerotic lesions^20–23^. Quantitative flow ratio (QFR) is a recent coronary physiological assessment that estimates a virtual FFR value on a three-dimensional model of the coronary artery, derived from two invasive angiographic views in end-diastolic frames^9,24,25^. To have patient-specific QFR, an approximation of contrast velocity through frame count analysis is derived. Contrast velocity then is estimated through frames of contrast entry and exit within the vessel segment and facilitates the computation of pressure drop along the vessel^26–28^. However, for a precise simulation of the flow patterns within vessels, it is vital to have a comprehensive depiction of each patient’s boundary conditions (BCs). This can be achieved by employing lumped parameter models to calculate and apply microvascular resistance values, ensuring accurate estimation of outlet conditions^5,29,30^. Some investigations projected pulse pressure using the two-element Windkessel model, a methodology that enhances the simulation of the aorta and arterial walls’ flexibility and deformability^13,31^. Nonetheless, obtaining the pulsating aortic pressure necessitates the insertion of a pressure wire into the aorta, which contradicts the objective of obtaining a noninvasive FFR instead of directly measuring FFR through invasive means.

Therefore, due to the lack of a comprehensive computational model to address the above-mentioned matters, the main goal of this study is to establish an efficient and highly accurate proof-of-concept framework for non-invasive FFR prediction and haemodynamic distribution throughout the 3D vessel to identify early vulnerable plaques. To be precise, five 3D patient-specific geometries are created using 2D angiography images via image segmentation algorithms. Following this, non-invasive haemodynamic metrics such as Time-Averaged Wall Shear Stress (TAWSS), Oscillatory Shear Index (OSI), and Residence Ratio Time (RRT) that influence disease progression are investigated and compared for each case. Moreover, four different BCs are applied, and the differences in haemodynamic parameters prediction in simulations are quantified afterward. At this point, a three-element Windkessel model at the outlet with average parameters and an average pressure wave at the inlet simulating the induced hyperemia within the patient’s distal microvascular system is applied to have accurate non-invasive BCs.

## Method

### Geometry

**Figure 1:**
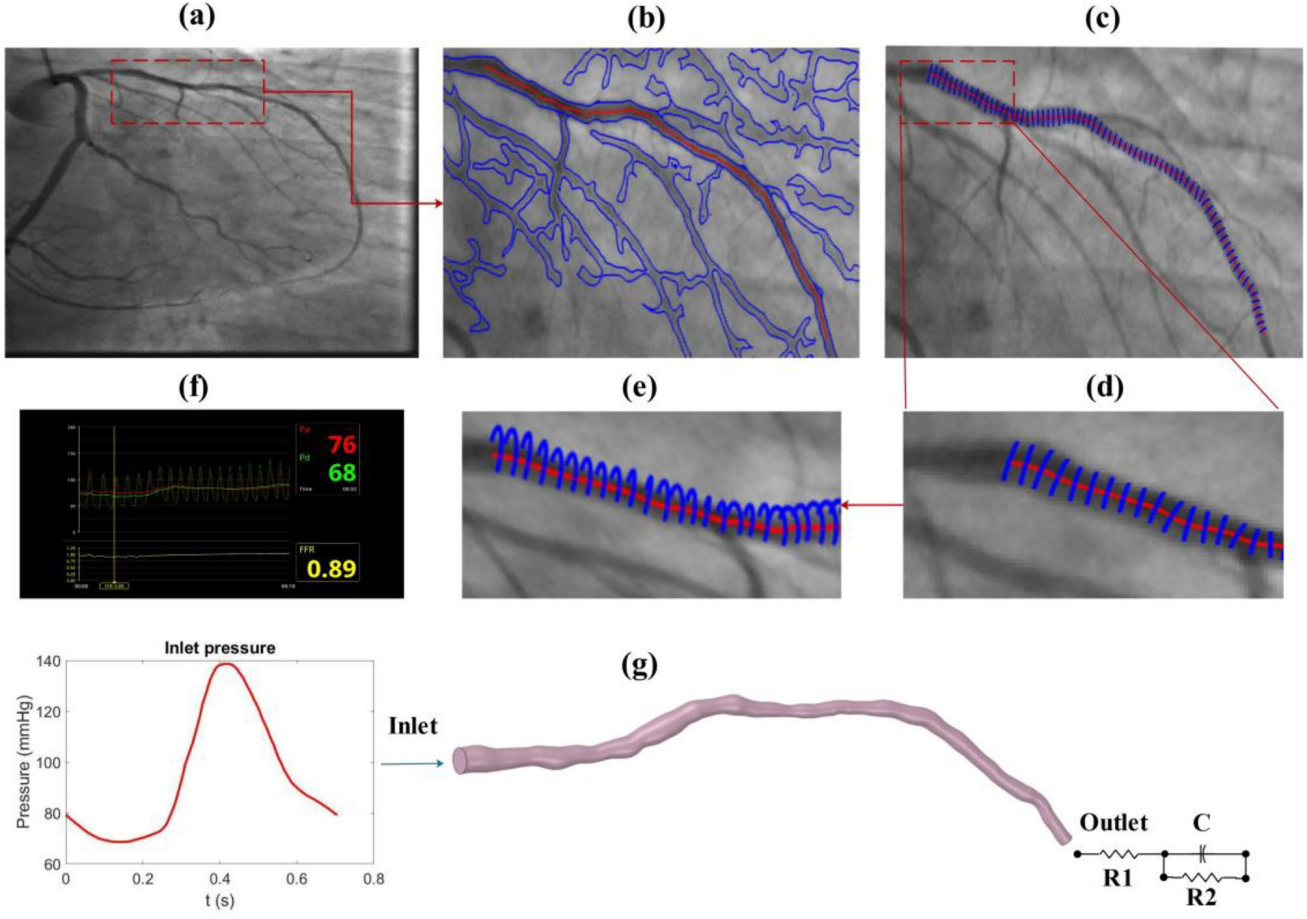
Schematic of the presented study. (**a**) Targeted vessel. (**b**) Edge detection, (**c**) 2D to 3D reconstruction, (**e**, **d**) continuous revolving technique, (**f**) Invasive pressure wave, and (**g**) 3D geometry with average transient pressure derived from invasive FFR measurements among five patients at the inlet and 3WKM at the outlet.

Three-dimensional (3D) blood vessel domains are constructed from 2D angiography images of five patients who underwent the angiography procedure. The research protocol received approval from the ethics committee at the Rajaie Cardiovascular Medical and Research Center and the participant signed informed consent and all methods were performed under the relevant guidelines and regulations. FFR is commonly practiced to be calculated and evaluated during the diastolic phase of a cardiac cycle. Therefore, one of the highest intensity frames during the end-diastolic phase wherein the maximum stenosis can be seen in the targeted region (vessel) must be selected as shown in Fig. 1a. Upon successful selection of the appropriate frame, image processing algorithms are eventually utilized to detect the vessel’s edge (vessel wall) and centerline from a 2D angiography image via MATLAB (R2019a version, Mathworks, Natick, USA) demonstrated in Fig. 1b. Therefore, to create a 3D domain, an edge-to-edge continuous revolving technique is appointed wherein all other diastole frames are read and coupled to better approximate the estimated reconstructed 3D blood domain (Fig. 1c, e). One of the crucial stages of image processing is to detect the edge and centerline as accurately as possible since each pixel is vital in having an accurate approximation of the final geometry, this is to boost the domain accuracy as the over and under estimation of these techniques has direct effect on pressure drop and thus the FFR value. For this purpose, the unique threshold value used herein is tuned and modified due to the variety of grayscale value ranges during the segmentation method; these values are varied throughout all angiography images. This is to eradicate any imaging flaws that may occur in capturing each frame and is eventually normalized by considering all frames during the entire cardiac cycle. A unique edge-to-edge continuous revolving technique is appointed that connects and correlates with each pixel and the eventual one step ahead of the cardiac step pixel to connect the continuous cross-sectional area and minimize the geometry error and unlikeliness (Fig. 1d, e). The correct elimination of the side branches is vital, a unique technique is prescribed to intelligently detect and cut the unnecessary regions that can lead to an incorrect assumption of blood flow proportion and behavior. Eliminating these branches requires more realistic BCs that account for these areas such as Windkessel models wherein the correct values of the 0D parameters can account for compromised regions. The acquired geometry is processed through other imaging techniques for the whole cardiac cycle to validate the vessel at each frame taken during the angiography. To have a more accurate approximation of haemodynamic parameters, the 3D domains must be divided into small sections. This step is so called meshing; hence, the 3D domain is imported into ANSYS-ICEM 20.1 (ANSYS Inc., PA, USA) for discretising the domain into smaller elements (Meshing)*.* The number of nodes and tetrahedral cells in the geometry is almost 50,000 and 120,000, respectively for all patient-specific models. Seven prismatic layers with a 1.1 growth rate are used to reduce the computational errors near the wall region. The correct insertion of prismatic layers at the wall improves the calculation of the WSS parameters which are significant haemodynamic factors in coronary blood flow. The correct estimation of WSS parameters can assist clinicians with a better understanding of the disease state and its progression. The meshing technique has been chosen in such a way that along the stenosis region, a higher number of mesh elements are appointed with an elevated sensitivity around the targeted regions. Two more mesh elements were appointed and calculated for this study, however, the present mesh model discussed in this study showed promising results in terms of the percentage of error and clinic-friendly computational time. The governing equation for the blood flow based on the finite volume method is solved using ANSYS-CFX 20.1 (ANSYS Inc., PA, USA). The Sensitivity study is provided as follows in Table 1.

**Table 1:** Numerical sensitivity study.

Sensitivity study	Coarse cells number	Medium cells number	Fine cells number	Extra fine cell number	Coarse and medium error (%)	Medium and fine error (%)	Fine and extra fine error (%)
Patient 1	60,000	115,000	190,000	250,000	3	0.37	0.02
Patient 2	65,000	125,000	200,000	250,000	3.2	0.39	0.02
Patient 3	65,000	135,000	195,000	250,000	2.9	0.35	0.01
Patient 4	70,000	130,000	200,000	250,000	3.3	0.37	0.02
Patient 5	65,000	135,000	210,000	250,000	2.8	0.4	0.03

### Boundary conditions

In general, four methods of BCs are applied at each model’s boundaries. As discussed earlier, BCs are to mimic the upstream and downstream behavior of the flow for the inlet and outlet of the vessel, respectively. Otherwise one must include the entire geometry of the patient which is not computationally reasonable for these emergency cases. In addition, due to the negligible compliance characteristics of these vessels, it is a common practice to assume the walls of the models to be rigid. A constant steady proximal pressure (Pa) from invasive FFR measurement (Fig. 1f) at the inlet and a patient-specific resistance model at the outlet with the use of ‘Hagen–Poiseuille law’ in Eq. (1) is deployed (first method). The ‘Hagen–Poiseuille law’ states the pressure drop in terms of the flow rate:

1 $$\Delta P = RQ$$

where R is the hydrodynamic resistance. This is carried out by discretizing the fluid domain into small elements and finding the solution for every element in the domain.

The other approach is an invasive transient pressure and patient-specific three-element Windkessel model (3WKM) at the inlet and outlet are applied, respectively. At the inlet, a cycle wherein FFR is measured in invasive proximal pressure measurement (Fig. 1f), exported, and applied as the inlet BC (Fig. 1g). The 3WKM is deployed at the outlet to provide realistic dynamic BC. In this model, as provided in equation Eq. (2), Pressure (P) and blood flow (Q) correspond to voltage and current in a circuit, respectively using a hydraulic-electrical analogy (0 Dimensional) as shown in Fig. 1g (second method):

2 $$P = \left( {R_{1} + R_{2} } \right)Q - R_{2} C\frac{dP}{{dt}} + R_{1} R_{2} C\frac{dQ}{{dt}}$$

wherein, $R_{1}$ is characteristic impedance, $R_{2}$ is hydrodynamic resistance and C is the compliance of the patient’s vessel. To tune 3WK parameters, distal pressure from invasive angiography measurement is used to minimize the error between the pressure wave obtained from 3WKM and distal invasive pressure. Wherein Windkessel parameters are adjusted through the process of curve fitting to minimize the disparity between measured and anticipated pressure values, accurate estimation of these parameters is achieved^32^.

Additionally, the average of invasive transient pressures and the 3WKM with average parameters at the inlet and outlet are utilized, respectively (third method). The inlet pressure for each patient has the same pattern. Therefore, the mean pressure at the inlet is scaled by the specified pressure of one of the patients, Meanwhile, the domain is obtained by averaging the transient pressures across all patients using the following technique:

1. Appling FFT (Fast Fourier Transformation): Computing the FFT of each signal to convert them into the frequency domain.
2. Averaging the Spectra: Adding the FFT results of the signals together element-wise and then dividing by the total number of signals.
3. Inversing FFT: Converting the averaged FFT back to the time domain using the Inverse FFT.

At the outlet, each Windkessel parameter is achieved from the average of patient-specific parameters among all patients.

Eventually, in the fourth method, invasive transient pressures (Actual transient BCs) obtained through invasive FFR measurements are applied at the inlet and outlet (patient-specific BCs). All BCs rely on invasive data except for average BCs which is the third method. Additionally, for all models, a rigid wall with a no-slip BC is assumed. Table 2 presents a comprehensive summary of all the methods used to define BCs:

**Table 2:** A classification of methodologies for defining BCs.

Methodology	Inlet	Outlet	Wall
1	Constant steady pressure	Patient-specific R	Rigid
2	Actual transient pressure	Patient-specific RCR	Rigid
3	Average transient pressure	Average RCR	Rigid
4	Actual transient pressure	Actual transient pressure	Rigid

The second-order backward Euler scheme with a time step of 0.005 s is used to discretise the governing equations, and the maximum residual mean square errors are set to $1\times {10}^{-5}$. The continuity and Navier–Stokes equations (Eqs. (3) and (4), respectively) were solved:

3 $$\nabla \cdot \vec{u} = 0$$

4 $$\rho \frac{{\partial \vec{u}}}{\partial t} + \rho \left( {\vec{u} \cdot \nabla } \right)\vec{u} + \nabla p - \mu \Delta \vec{u} = 0$$

where $\overrightarrow{u} , \rho , p$ and $\mu$ represent the fluid velocity vector, density, pressure, and dynamic viscosity, respectively. Blood is assumed to be incompressible fluid with a density of 1056 kg $/{m}^{3}$. Blood is simulated as a non-Newtonian fluid because its shear-thinning effect is significant in small vessels^19^. The power law, Carreau, and Carreau-Yasuda models make very similar predictions in left coronary arteries in all situations^19^. Therefore, the Carreau–Yasuda model is applied to calculate the fluid's viscosity in models as shown in Eq. (5). Where $\mu$ is viscosity, $\gamma$ is shear rate, a is Yasuda exponent, and m is Carreau–Yasuda Power Law Index${, \mu }_{0}, {\mu }_{\infty } and {\lambda }_{CY}$ are, Carreau–Yasuda zero shear viscosity, infinite shear viscosity and time constant, respectively provided in Table 3:

5 $${\upmu } = \left( { {\upmu }_{0} - {\upmu }_{\infty } } \right)\left( {1 + \left( {{\uplambda }_{{{\text{CY}}}} \dot{\gamma }} \right)^{a} } \right)^{{\left( {{\text{m}} - {1}} \right)/{\text{a}}}} + {\upmu }_{\infty }$$

**Table 3:** Parameters of Carreau-Yasuda blood viscosity model.

$\mu_{\infty }$	$\lambda_{CY}$	$m$	${\text{a}}$	$\mu_{0}$
$0.0035{\text{ Pa}}{\text{.s}}$	$1.902{\text{ s }}$	$0.22$	$1.25$	$0.056 {\text{Pa}}{\text{.s}}$

The viscosity parameters applied in this investigation are established by Sandeep et al.^19^. Two cardiac cycles are applied to reach periodic repetitive states (periodic steady state). The third cycle is used to extract the results presented in this study. To post-processing the data, ANSYS CFD-Post and MATLAB (R2019a version, Mathworks, Natick, USA) are used.

**Figure 2:**
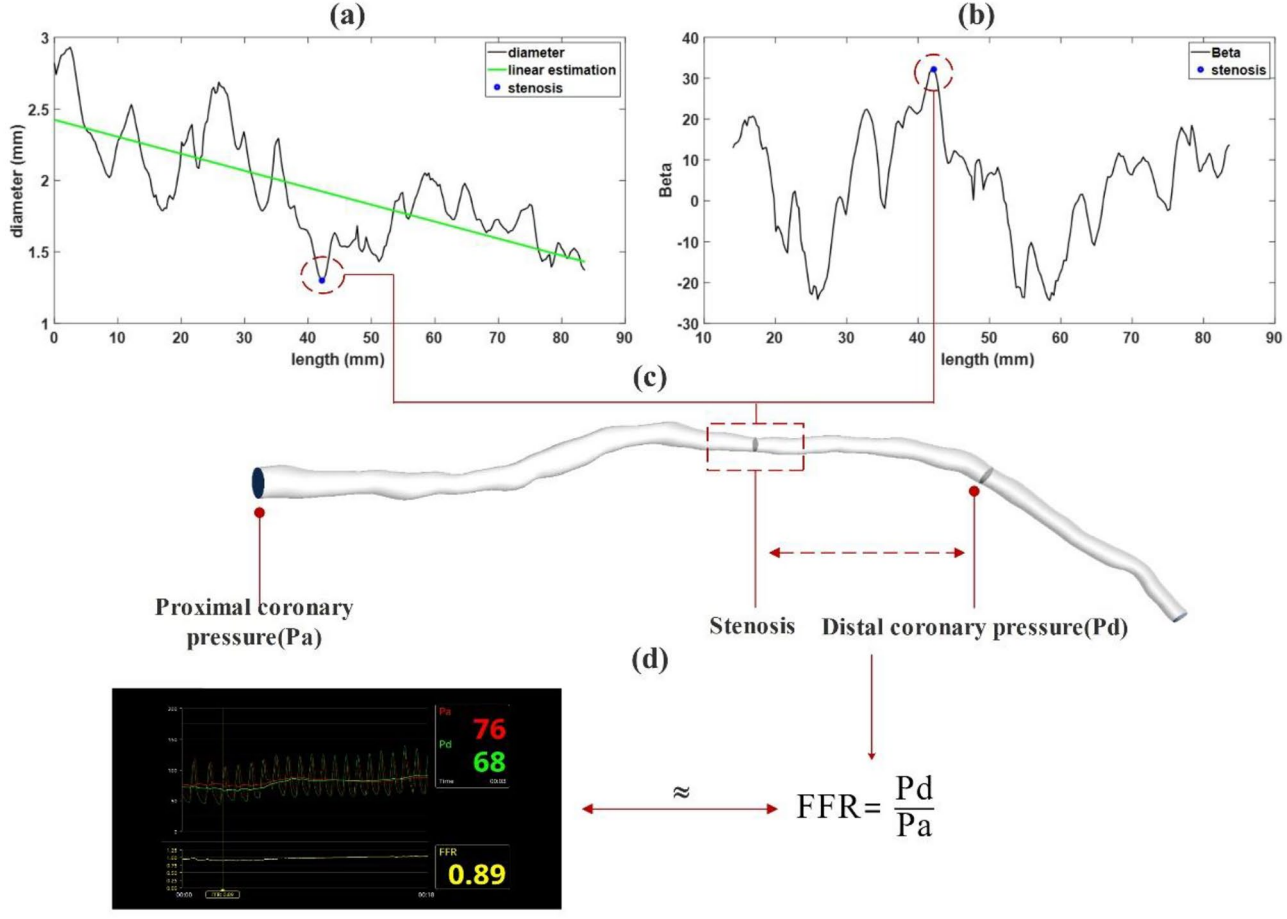
Calculation of FFR for specific time and location in the targeted vessel. (**a**) diameter versus length diagram, (**b**) $\beta$ versus length diagram, (**c**) Finding stenosis region and approximating location of distal pressure measurement in geometry, and (**d**) A comparative analysis of invasive FFR and virtual FFR.

The diagram in Fig. 2a demonstrates the diameter of the vessel against length, a linear function is fitted to show the slope of diameter reduction from upstream to downstream of the stenosis. The lowest point which is unexpected in the middle of the vessel shows a stenosis region, a further validation of the geometry reconstruction that provides a better understanding of each narrowed vessel. However, the stenosis region may be better detected in Fig. 2b, which showes $\beta$, a dimensionless parameter, versus length. $\beta$ is the degree of stenosis and is defined as a ratio of cross-sectional diameters: $\beta =100\%\times (1-d/D)$ , where d and D, are the diameter of the stenosis and the average of the cross-sectional diameters of upstream points of the narrowest region, respectively, which is another validation for the most narrowed region for the created fluid domain. As can be seen in Fig. 2a, b, the stenosis region is collocated in length for each patient. To calculate the virtual FFR, the inlet pressure is assumed as proximal coronary pressure (Pa). The location of distal coronary pressure (Pd) is approximated to correspond to the wire position in each vessel as demonstrated in Fig. 2c. FFR, which is the ratio of distal coronary pressure to proximal coronary pressure, is calculated for each patient in Fig. 2d. Following this, each model undergoes a comparative analysis, contrasting virtual FFR against invasive FFR.

## Results

The study demonstrated and compared the results of four different BCs methods across a set of 20 models. The process of reconstructing a 3D model from angiographical images for a single patient typically takes around two minutes once the operator identifies the diastolic frame. However, the overall duration can vary between 3 ± 1 min, depending on the operator. Moreover, this study was conducted on a Windows 10 workstation with a 3.5 GHz CPU and 64 GB RAM. The CFD simulation takes 10 min for a transient pressure at the inlet and 3WKM at the outlet model, for each patient.

### Pressure and velocity distribution

**Figure 3:**
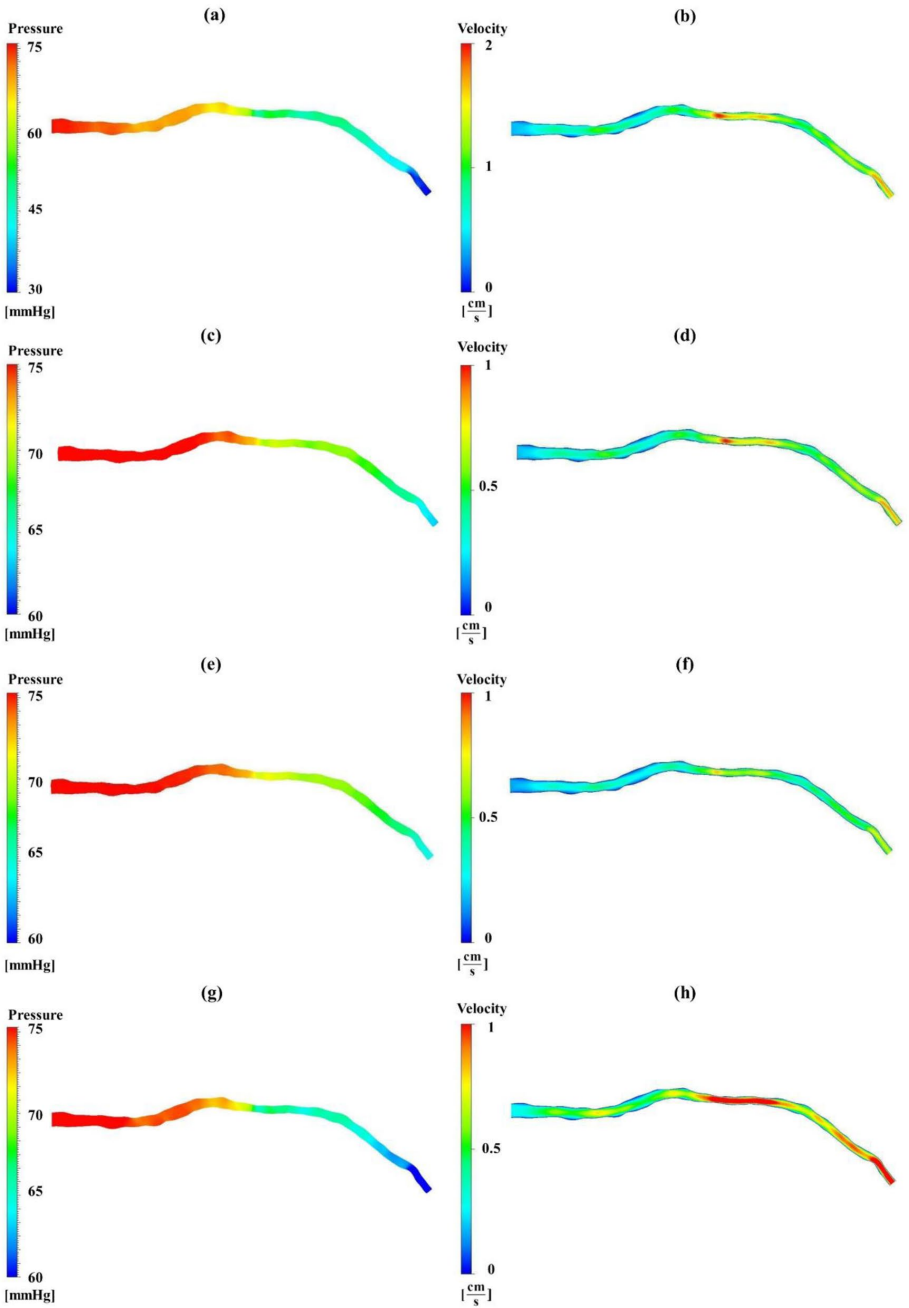
Pressure and velocity distribution. (**a**, **b**) Steady-state pressures from invasive FFR data at the inlet and Patient-specific resistance at the outlet, **(c**, **d**) Patient-specific transient pressure at the inlet, 3WKM with patient-specific parameters at the outlet, (**e**, **f**) Average transient pressure at the inlet, 3WKM with average parameters at the outlet, and (**g**, **h**) real-time transient pressures acquired from invasive FFR measurements at both the inlet and outlet.

Figure 3 provides information about the pressure and velocity distribution under these BCs for each patient. Notably, all cases exhibit higher pressure values before the stenosis, followed by a decline right after the stenosis. Furthermore, high velocity within the stenosis and near the outlet can be seen due to a reduction in diameter. There is a striking similarity in the pressure pattern and almost identical pressure values observed between Fig. 3c, e which are patient-specific BCs and average BCs, respectively. Among two out of four models in Fig. 3c, e, a consistent pressure drop of 15 [mmHg] is observed, resulting in pressures ranging from 75 [mmHg] at the inlet to 60 [mmHg] near the outlet. However, Fig. 3a, utilizing steady-state BCs, depicts a markedly higher pressure drop, with pressure declining from 75 [mmHg] at the inlet to 30 [mmHg] near the outlet.

In terms of velocity, the higher pressure drop has resulted in elevated velocity values, particularly evident in the steady-state BCs (Fig. 3b), where the stenosis region reaches its peak velocity at 2 [m/s] which is double in other cases. Despite a significant 25% rise in velocity observed for the patient-specific model at the stenosis region compared to average BCs, the pressure drop is almost the same between patient-specific and average BCs. This finding underscores the importance and similarity of these two models (Fig. 3d, f). Additionally, Fig. 3h, which has invasive transient pressure BCs, shows higher velocity values just after the stenosis relative to the other models.

**Figure 4:**
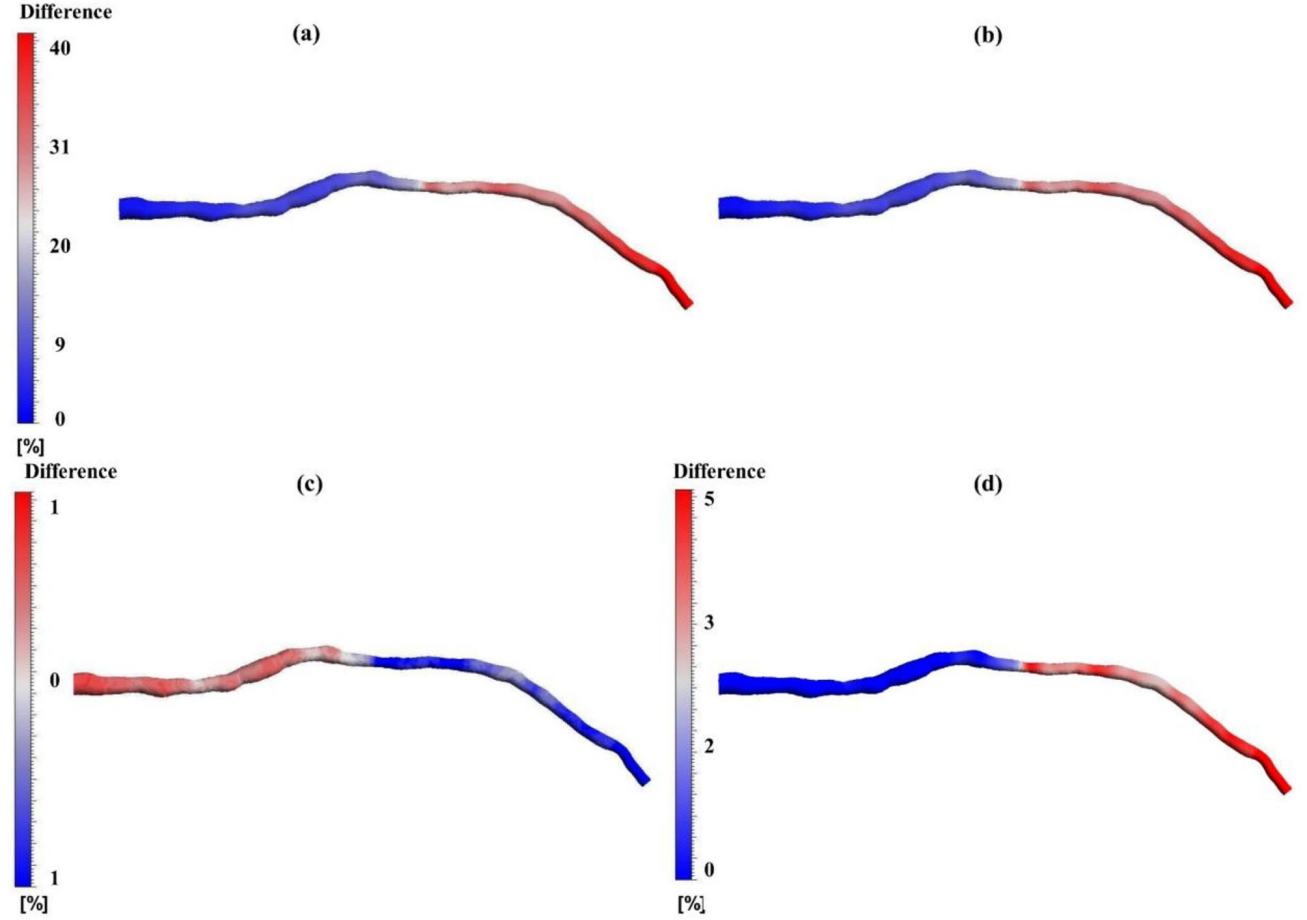
The percentage difference in pressure at the FFR time point for one of the patients using three color scales: (**a**) The difference between patient-specific and steady-state BCs models, (**b**) the difference between average and steady-state BCs models, (**c**) the difference between patient-specific and average BCs models, and (**d**) the difference between patient-specific and actual transient pressure BCs models.

In Fig. 4, the percentage difference in pressure is depicted across four distinct BC models for one of the patients. Specifically, Fig. 4a, b provide a comparison of pressure distribution between the patient-specific and average BC models with the steady-state BC model, respectively. Both figures illustrate a similar difference pattern and values, indicating that the pressure values near the outlet are underestimated in the steady-state model, highlighting the importance of the outlet BC. The difference in Fig. 4a, b starts from 0% at the inlet and rises toward the outlet of the geometry, eventually reaching a 40% difference. Moreover, Fig. 4c presents the pressure difference between patient-specific and average BCs, showing a remarkable similarity where the absolute error is less than 1%. To be precise, the patient-specific BCs model exhibits 1% higher pressure at the inlet and 1% lower pressure at the outlet compared to the average BCs model. Nonetheless, the pressure levels are identical just before the stenosis region in both models. Figure 4d presents the difference between patient-specific and actual transient pressure BC models. It can be seen that the difference is less than 2.5% before the stenosis, but it doubles near the outlet, revealing that patient-specific displays less pressure drop and higher pressure values along the geometry compared to actual transient pressure. Overall, these findings emphasize the importance of considering BCs in modeling to ensure accurate results.

### Computed FFR versus Invasive FFR

The accuracy of virtual FFR derived from simulations is investigated by comparing it with invasive FFR data across 10 models. The findings of these comparisons are presented in Tables 4 and 5, illustrating the agreement between invasive and virtual FFR under patient-specific and average BCs, respectively. Across all simulations, the observed errors between invasive and virtual FFR were found to be less than 5%. Furthermore, Table 4 provides patient-specific three-element Windkessel parameters for each patient, accompanied by their corresponding $\beta$ values, which range from 18 to 45, indicating intermediate blockage. Table 5 shows the absolute percentage difference(error) between the patient-specific and average three-element Windkessel parameters.

**Table 4:** Comparison of the invasive FFR and virtual FFR with patient-specific BCs.

Patients	Invasive FFR	Virtual FFR	$\beta$ % of stenosis	R1 (mmHg s ml^−1)	R2 (mmHg s ml^−1)	C (ml mmHg^−1)	FFR error (%)
Patient 1	0.89	0.885	32	0.39713	163.28846	0.00357	0.5
Patient 2	0.85	0.87	20	0.21380	76.02368	0.00355	2.3
Patient 3	0.84	0.869	18	0.335892	151.69549	0.00255	3.4
Patient 4	0.89	0.90	45	0.10599	68.12693	0.00354	1.1
Patient 5	0.86	0.862	36	0.25837	128.63917	0.00297	0.2

**Table 5:** Comparison of the invasive FFR and virtual FFR with average BCs.

R1 (mmHg s ml^−1) = 0.2622364, R2 (mmHg s ml^−1) = 117.554746, C (ml mmHg^−1) = 0.003236						
Patients	Invasive FFR	Virtual FFR	FFR error (%)	R1 error (PS, Average) (%)	R2 error (PS, Average) (%)	C error (PS, Average) (%)
Patient 1	0.89	0.892	0.2	33	28	9
Patient 2	0.85	0.87	2.3	22	54	8
Patient 3	0.84	0.85	1.1	21	22	26
Patient 4	0.89	0.93	4.4	147	72	8
Patient 5	0.86	0.87	1.1	1	8	8

### Time average wall shear stress

TAWSS (Time-Averaged Wall Shear Stress) is a critical parameter that characterizes the dynamic stress exerted on the blood vessel walls during the cardiac cycle (T). It is obtained by calculating the average WSS over the entire cardiac cycle. The formula for computing TAWSS is given in Eq. (6).

6 $$TAWSS = \frac{1}{T}\mathop \int \limits_{0}^{T} \left| {\overrightarrow {WSS\left( t \right)} } \right|dt$$

**Figure 5:**
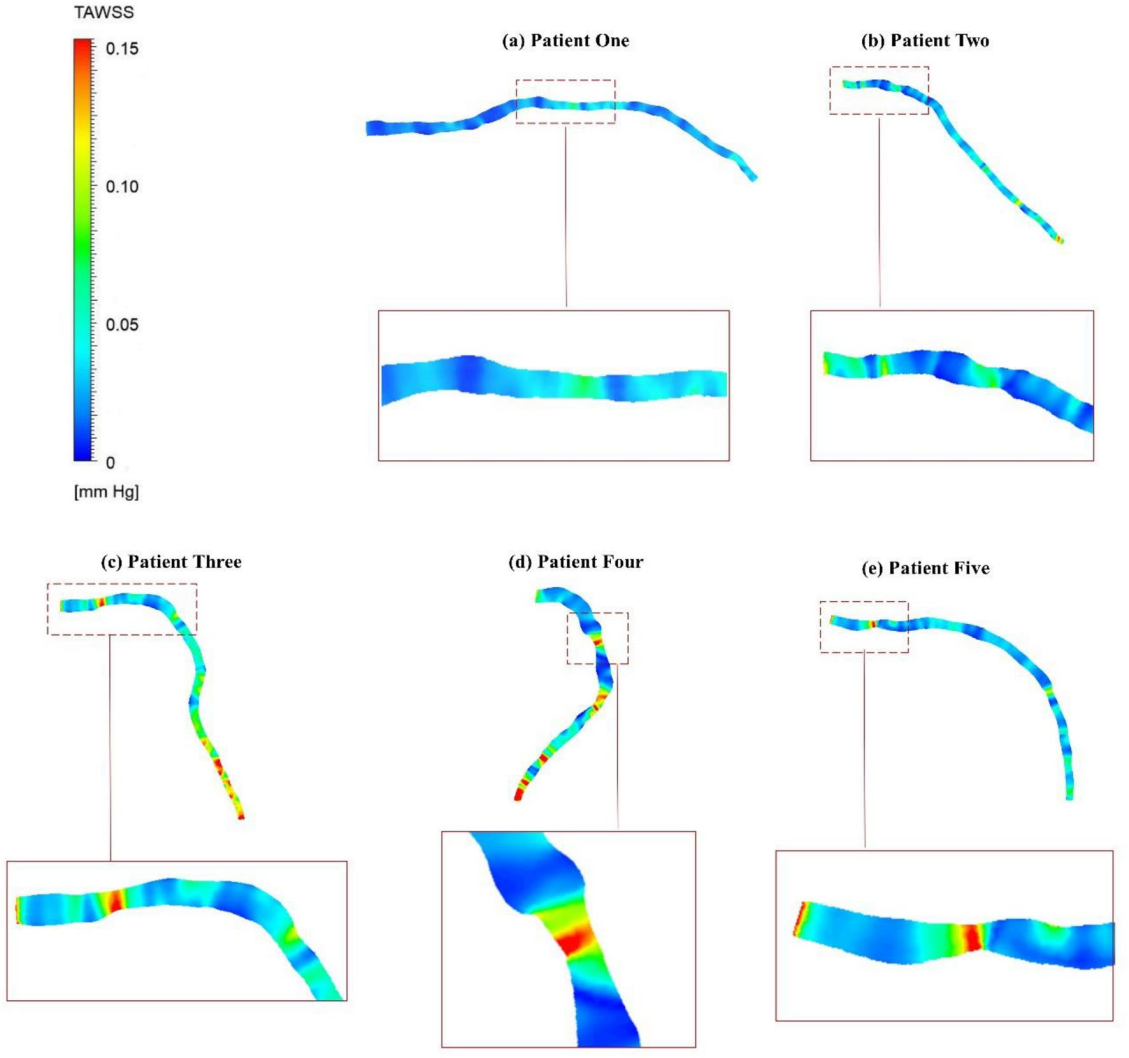
TAWSS distribution for patient-specific BCs simulation.

Figure 5 illustrates the distribution of TAWSS obtained from simulations conducted on five patients. The stenosis section exhibits higher TAWSS magnitudes, likely due to the elevated velocity magnitude compared to its adjacent areas. Furthermore, a significant increase in TAWSS is observed from the upstream to the downstream regions of the models, which reflects a probable area for plaque formation.In particular, in patients three, four, and five (Fig. 5c, d, e), the TAWSS value almost triples in the stenosis and downstream regions, reaching up to 0.15 [mmHg], while maintaining an approximate value of 0.05 [mmHg] in other regions. In contrast, for patients one and two (Fig. 5a, b), the TAWSS values demonstrate a less pronounced increase, almost doubling (0.1 [mmHg]) in the narrow regions.

### Oscillatory shear index

The Oscillatory Shear Index (OSI) is a metric used to quantify alterations in both the direction and magnitude of WSS experienced by endothelial cells. Its numerical range spans from 0 to 0.5, where a value of 0 signifies one-way WSS forces, while higher values indicate unpredictable changes in WSS force directions. Though OSI effectively captures WSS fluctuations during the cardiac cycle, it does not account for WSS magnitude. As a result, it is frequently complemented by TAWSS to provide a more comprehensive analysis of WSS patterns. The Eq. (7) is to calculate OSI as follows:

7 $$OSI = \frac{1}{2}\left( {1 - \frac{{\left| {\frac{1}{T}\mathop \smallint \nolimits_{0}^{T} \overrightarrow {WSS\left( t \right)} dt} \right|}}{TAWSS}} \right)$$

**Figure 6:**
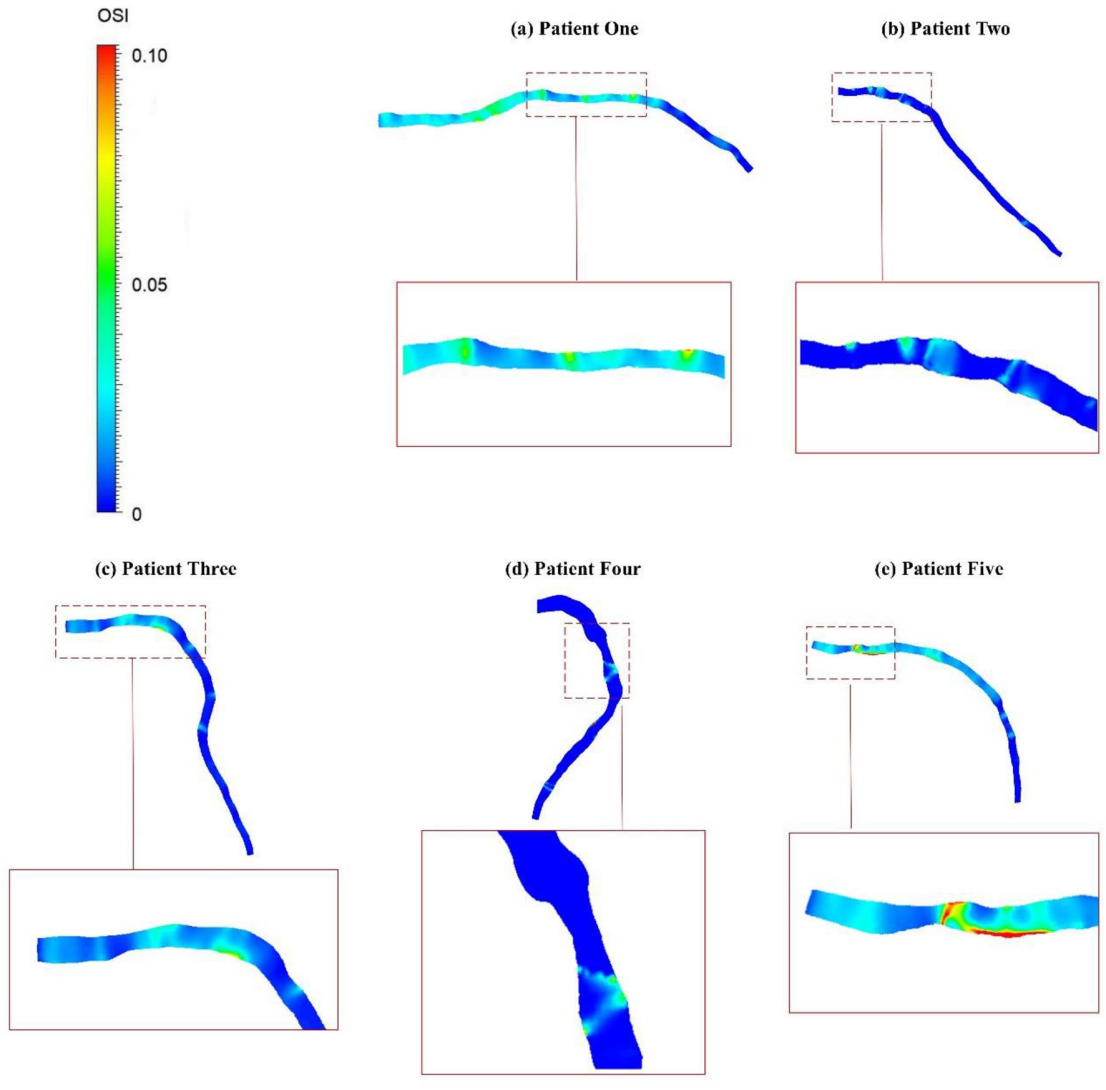
OSI distribution for patient-specific BCs simulation.

Figure 6 illustrates the OSI distribution for simulations utilising patient-specific BCs. Notably, high OSI values are observed just after the stenosis section, indicating a significant variation in blood flow direction compared to the mean flow in that region. Particularly, patients two, four, and five (Fig. 6b, d, e) adjacent to the stenosis with low TAWSS exhibit helical sections of high OSI. Particularly in Fig. 6e, the OSI value almost doubles and hits a peak after the stenosis. For patient one in Fig. 6a, the OSI increases by approximately 0.05 both before and after the stenosis, where low TAWSS occurs due to flow recirculation, creating a potential area for plaque formation. Similarly, the same phenomenon is observed for patient three in Fig. 6c, but with a slightly lower increase of just under 0.05.

### Residence ratio time

**Figure 7:**
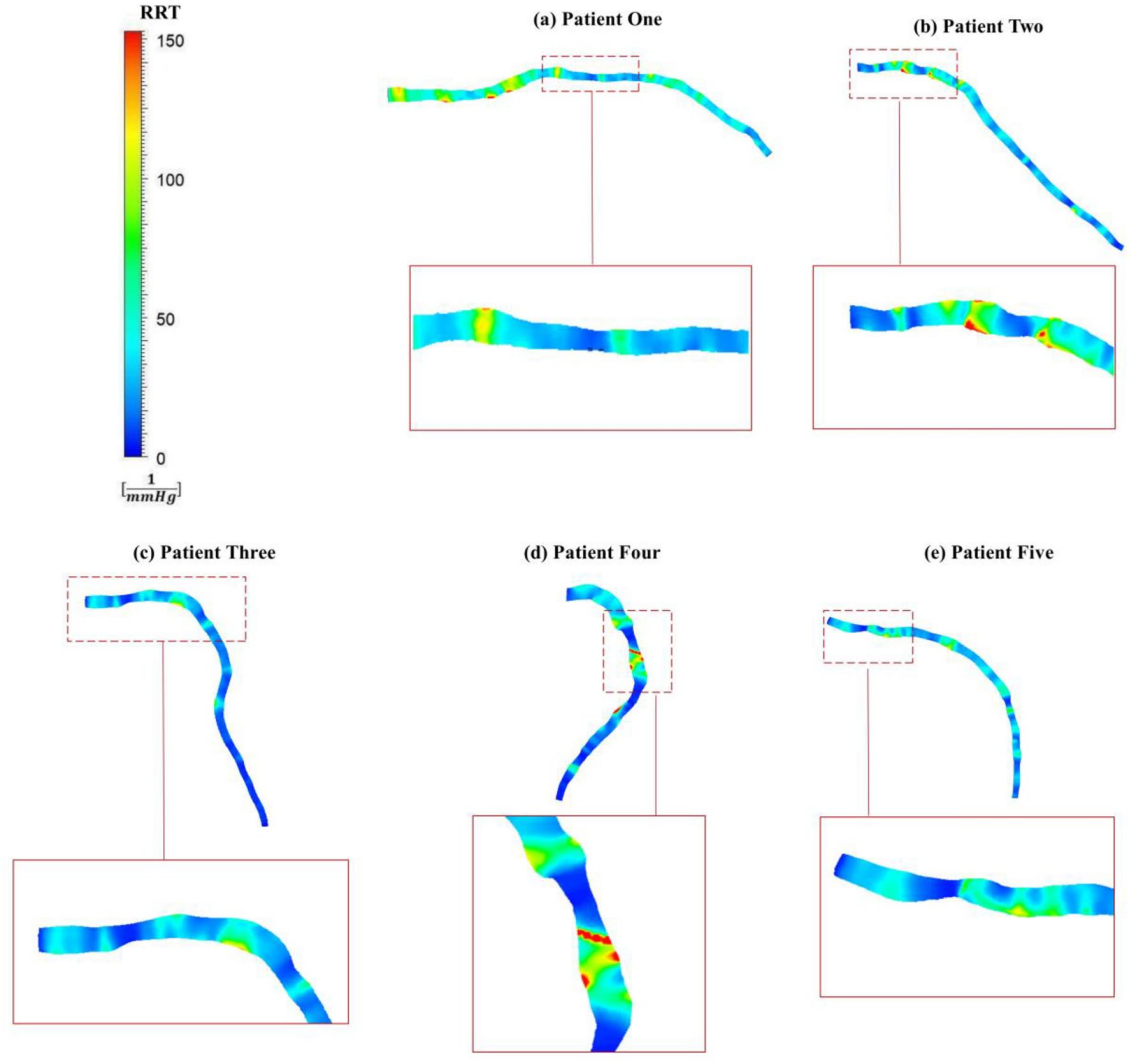
RRT distribution for patient-specific BCs simulation.

RRT (Residence Ratio Time) is a metric used to calculate the duration of a particle's stay adjacent to the vascular endothelium. This characteristic exhibits an adverse correlation with the size of the TAWSS vector and can vary between 0 and infinity. The contours of RRT for five patients are displayed in Fig. 7. The Eq. (8) is used to calculate RRT as follows:

8 $$RRT = \frac{1}{{\left( {1 - 2 \cdot OSI} \right) \cdot TAWSS}}$$

RRT demonstrates an increase both before and after the stenosis, showing a significant correlation with the OSI distribution in Fig. 6. Particularly, in the vicinity of the stenosis, helical sections with high OSI values correspond to elevated RRT values. This relationship is particularly prominent in patients two and four (Fig. 7b, d), where RRT values are nearly 150 [${\text{mmHg}}^{-1}$] higher compared to the stenosis regions. A similar trend is observed in patients three and five (Figs. 7c and 7e), albeit with a comparatively lower increase of approximately 50 to 100 [${\text{mmHg}}^{-1}$]. In the downstream region of patient three (Fig. 7c), the RRT value is almost zero due to the lack of significant changes in the cross-section, and the vessel maintains an almost constant radius.

### Percentage difference

**Figure 8:**
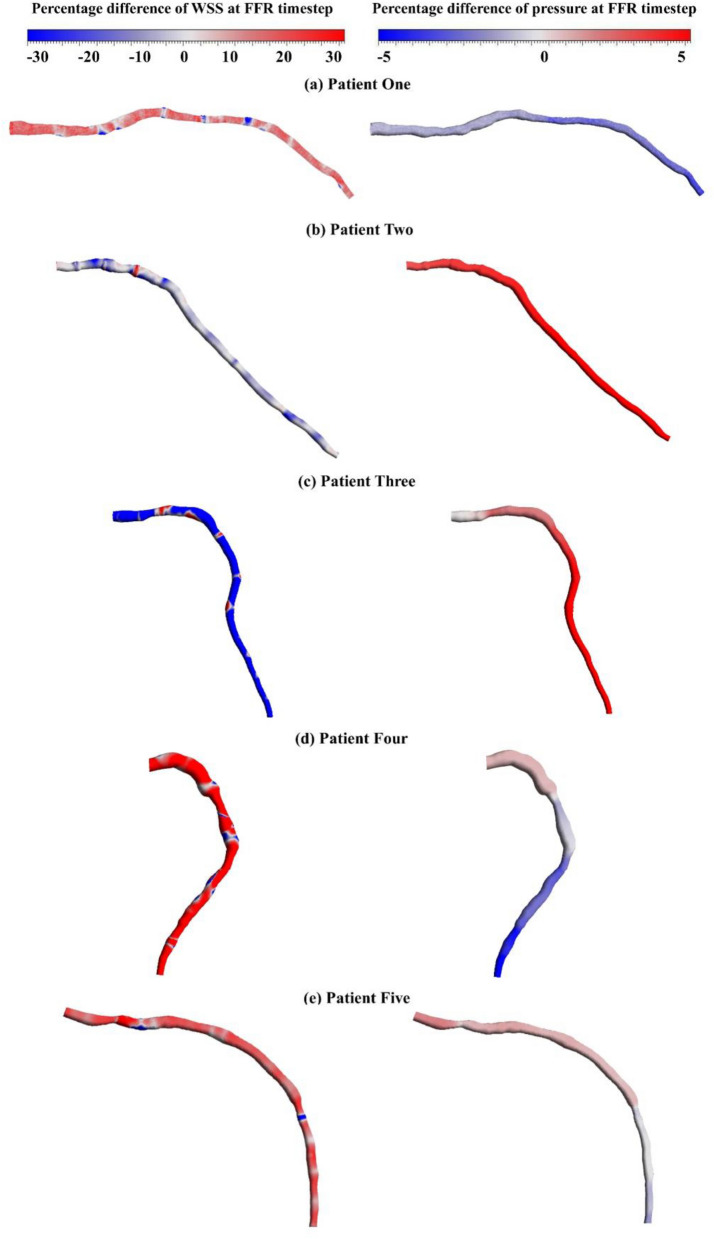
The percentage difference of pressure and WSS at the FFR time point for all patients comparing patient-specific BCs and average BCs.

Figure 8 demonstrates the percentage difference of pressure and WSS between two methods of patient-specific BCs and average BCs for five patients. The difference in WSS distribution throughout the vessel, especially after the stenosis region is significant. Patient three in Fig. 8c has the lowest WSS difference among other patients, which is the result of more similarity between pressure waves at the inlet and Windkessel parameters at the outlet in the two methods. Furthermore, the pressure difference between the two methods is shown. In most cases, the average BC overestimates pressure downstream except for patients two and three (Fig. 8b, c) which the average BC underestimates the pressure distribution.

## Discussion

This study introduces an innovative approach to virtually calculate FFR and investigate WSS haemodynamic indices in coronary arteries. Five patients with intermediate stenosis in the left anterior descending (LAD) are enrolled. Then 3D patient-specific geometries are efficiently constructed between 3 ± 1 min from 2D angiography images, representing an innovative and time-saving approach compared to previous studies that relied on intravascular ultrasound, X-ray angiography, or CTCA imaging for non-invasive FFR measurements^11,33–35^. Prior research has indicated differences in instantaneous WSS values between rigid and fluid–structure interaction (FSI) models, whereas, there is a similarity in TAWSS values between these two approaches^1,36–39^. Therefore, the simulations are deployed CFD with rigid walls to optimize computational efficiency. Moreover, non-Newtonian models predicted markedly higher pressure drop and shear stress throughout the cardiac cycle than the Newtonian model and led to more accurate pressure and shear stress distribution^19,40^. However, precise CFD simulations require patient-specific geometry and suitable BCs^18,41,42^. Four distinct BC methodologies are introduced and classified in Table 2. The first method, involving a constant steady pressure from proximal invasive measurements at the inlet and patient-specific resistance at the outlet, overestimates peak velocity in the stenosis region and pressure drop along the geometry. As a result, the FFR value predicted by this method is lower than invasive measurement, rendering it inaccurate despite its low computational cost.

The second method, utilizing invasive transient pressure at the inlet and patient-specific 3WKM at the outlet, demonstrates promising results in FFR prediction (Table 4), with errors for all five patients below 4%. However, this method relies on invasive measurements. To assess the viability of non-invasive and accurate BCs for coronary artery simulations, the third method introduces average pressure wave and average 3WKM at the inlet and outlet, respectively. This model shows good results with the calculated virtual FFR validating well against invasive FFR for all patients demonstrated in Table 5, with an error below 5%. Our approach addresses the drawbacks of invasive FFR measurement used in all prior methods because it does not involve insertion of pressure wire. The computational time for both the patient-specific BCs model and the average BCs model is the same, each taking almost 10 min due to the identical inlet and outlet BCs types. Moreover, these models exhibit comparable accuracy, evidenced by the pressure differences provided in Figs. 4c and 8, both below 5% along the vessel.

Finally, to illustrate the significance of the 3WKM boundary condition at the outlet, the fourth method is introduced involving invasive transient pressure at the inlet and outlet. While the inlet BC is similar to the second method, the velocity post-stenosis is almost 25% higher in Fig. 3h. Furthermore, Fig. 4d reveals a 5% difference in predicted pressure, indicating that this model is less accurate in predicting FFR value compared to the second and third methods. This comparative analysis underscores the critical role of an appropriate outlet boundary condition as it is capable of predicting the pressure/flow relationships in the arteries with a reasonable degree of accuracy^32^. As highlighted in prior research, the maximum pressure drop occurs near severe stenosis regions, and there is an elevated velocity of flow within constrained segments^14,22,34,43^. These findings are consistently depicted in Fig. 3 across various BCs.

FFR offers critical insights into the quantitative impact of stenosis on blood flow and the functional significance of stenotic lesions. In contrast, TAWSS provides valuable information on the mechanical stresses exerted by blood flow on arterial walls, highlighting areas of potential disturbance in blood flow patterns. The integration of FFR and TAWSS has a more comprehensive understanding of the physiological and biomechanical aspects of CAD, thereby enhancing the diagnostic and prognostic capabilities of cardiovascular assessment.

The haemodynamic parameters of TAWSS, OSI, and RRT are investigated in this study to show complex dynamics within coronary arteries (HOLMES value description and contours for all patients are presented in Supplementary Material). The TAWSS patterns in Fig. 5 align with established literature^1,21,44^, indicating elevated WSS at luminal narrowing areas and decreased WSS immediately following mild to high stenosis. Moreover, The high OSI values (Fig. 6) particularly after the stenosis section highlight areas prone to disturbed flow, align with findings in preceding investigations^14,45–47^, and underscore the substantial alteration in coronary artery blood flow direction relative to the mean flow in this specific region. Previous studies have also demonstrated elevated RRT in regions characterized by reduced WSS^45,46^, which indicates regions prone to atherosclerotic plaque calcification^48^. It is evident that areas preceding the stenosis region and immediately following the narrowing are susceptible to plaque development^49–51^.

## Conclusion

The current study has developed a robust and precise proof-of-concept framework for the non-invasive prediction of FFR and the characterization of haemodynamics within 3D vessels, facilitating the early identification of vulnerable plaques. The generation of patient-specific 3D geometries from 2D angiography images is coupled with the implementation of four distinct BCs methodologies to establish a reliable and accurate framework. Among these models, the average BCs model demonstrates promising results in predicting FFR, as well as providing accurate pressure and velocity contours. The computational time for geometry construction, combined with this BCs model, requires less than 15 min to generate results, eliminating the necessity for invasive pressure measurements. The observed similarity in FFR values and simulation results between the patient-specific and average boundary conditions indicates that by using the average BCs model, the patient-specific data for each patient may no longer be imperative for hospital application. Furthermore, haemodynamic-based indices have demonstrated effectiveness in enhancing the early detection and predictive accuracy of CAD. Therefore, a comprehensive analysis linking these haemodynamic parameters with virtual FFR enhances the diagnostic capabilities in plaque development.

## Limitations and future remarks

Our study specifically concentrated on patients with a measured FFR higher than 0.8. Unfortunately, resource constraints limited our capacity to include patients with FFR values below 0.7 in the current project and to improve the generalizability of our findings. Therefore, our study should be considered as a proof-of-concept framework. To enhance the comprehensiveness of our framework’s validation in the future, there is a need to extend the investigation to include patients with higher variations in FFR values.

### Supplementary Information

Supplementary Information.

## Data Availability

Correspondence and requests for materials should be addressed to S.E.P. and M.A. on reasonable request.
