# Supplementary material for: Non-invasive fractional flow reserve estimation in coronary arteries using angiographic images
Source: Sci Rep. 2024 Jul 8;14:15640. doi: 10.1038/s41598-024-65626-9 (PMC11231276; doi:10.1038/s41598-024-65626-9)
Supplement: Supplementary file 1 — Supplementary Information. [file 41598_2024_65626_MOESM1_ESM.docx]

**Non-invasive fractional flow reserve estimation in coronary arteries using angiographic images**

Hadis Edrisnia ^1^, Mohammad Hosein Sarkhosh^2^, Bahram Mohebbi MD^3^, Seyed Ehsan Parhizgar MD^3, *^, Mona Alimohammadi^1^

^1^ Department of Mechanical Engineering K. N. Toosi Univeristy of Technology, Tehran, Iran

^2^Department of Mechanical Engineering, Sharif University of Technology, Tehran, Iran

^3^Rajaie Cardiovascular, Medical and Research Center, Iran University of Medical Sciences, Tehran, Iran

^*^ Corresponding author: [separhizgar@gmail.com](mailto:separhizgar@gmail.com)

The PDF file includes:

Text

Fig. S1. HOLMES distribution for patient-specific BCs simulation

**Highly Oscillatory, Low Magnitude Shear**. Highly oscillatory, low magnitude shear (HOLMES) is an insightful index that enhances comprehension and establishes a connection between OSI and TAWSS. Moreover, HOLMES serves as a valuable tool to identify regions with increased infiltration in low oscillatory zones. This parameter effectively reduces the index in areas exhibiting fluctuating and low WSS, providing valuable insights into the haemodynamic characteristics of such regions. The equation $($*8*) is to calculate HOLMES provided as follows:

| $HOLMES=TAWSS(0.5-OSI)$ | $($8$)$ |
| --- | --- |

Figure 7 illustrates the distribution of the HOLMES index for five patients, showing a strong correlation with the TAWSS distribution. As the severity of the stenosis increases, maximum HOLMES concentrations shift closer to the stenosis region. Notably, Figure 7 highlights locations with a high number of HOLMES, which correspond to regions exhibiting low OSI (Figure 6) and high TAWSS (Figure 5). In particular, Patients 3, 4, and 5 (Figure 7c,Figure *7*d, and Figure *7*e) experienced 50% higher HOLMES values near the stenosis compared to Patients 1 and 2 (Figure 7a and Figure *7*b). Additionally, Patients 3 and 4 displayed approximately 80% higher HOLMES values downstream of the vessel.

**
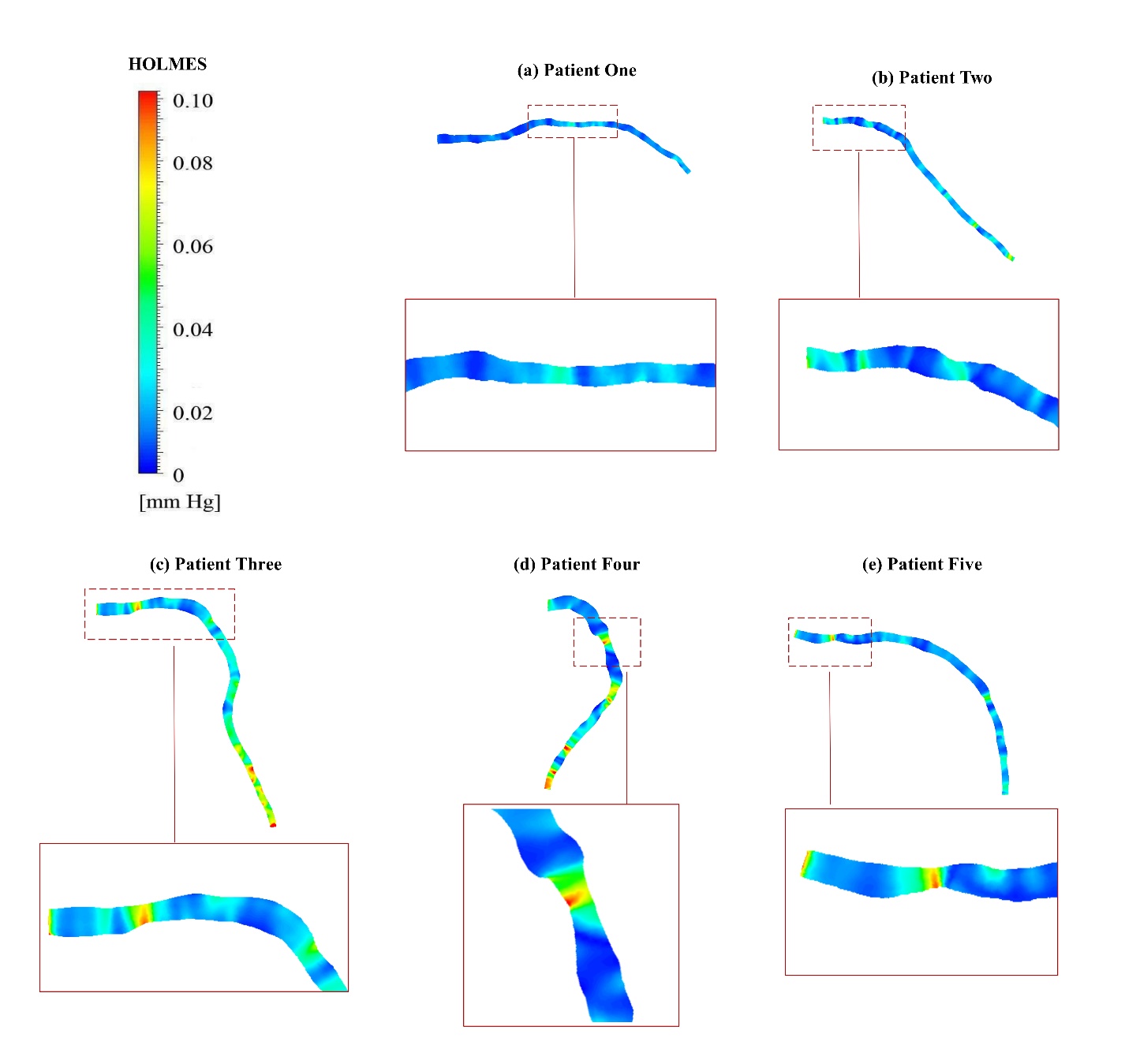
**

Figure S1. HOLMES distribution for patient-specific BCs simulation
